# Early Elevated IFNα Identified as the Key Mediator of HIV Pathogenesis and its low level a Hallmark of Elite Controllers

**DOI:** 10.21203/rs.3.rs-2813601/v1

**Published:** 2023-05-11

**Authors:** Hélène Le Buanec, Valérie Schiavon, Marine Merandet, Alexandre How-Kit, David Bergerat, Céline Fombellida-Lopez, Armand Bensussan, Jean-David Bouaziz, Arsène Burny, Gilles Darcis, Hongshuo Song, Mohammad M. Sajadi, Shyamasundaran Kottilil, Robert C. Gallo, Daniel Zagury

**Affiliations:** 1 Université de Paris; INSERM U976, HIPI Unit, Institut de Recherche Saint-Louis, F-75010 Paris, France.; 2 Laboratory for Genomics Foundation Jean Dausset-CEPH; Paris France; 3 Laboratory of Infectious Diseases, GIGA-I3, GIGA-Institute University of Liege; 4000 Liege, Belgium; 4 Dermatology Department, Hôpital Saint-Louis, Assistance Publique-Hôpitaux de Paris (AP-HP), Paris, France; 5 Laboratory of Molecular Biology, Gembloux Agrobiotech,University of Liège; Belgium; 6 Global Virus Network, Baltimore, MD 21201, USA.; 7 Institute of Human Virology, School of Medicine, University of Maryland; Baltimore MD, 21201, USA, Department of Medicine, School of Medicine, University of Maryland, Baltimore, MD, 21201, USA.; 8 University of Maryland School of Medicine; Baltimore, MD 21201, USA, Program in Oncology, Marlene and Stewart Greenebaum Comprehensive Cancer Center, University of Maryland, Baltimore, MD 21201, USA.; 9 21CBIO; Paris France.

## Abstract

Advances in HIV therapy came from understanding its replication. Further progress toward “functional cure” -no therapy needed as found in Elite Controllers (EC)- may come from insights in pathogenesis and avoidance by EC. Here we show that all immune cells from HIV-infected persons are impaired in non-EC, but not in EC. Since HIV infects few cell types, these results suggest an additional mediator of pathogenesis. We identify that mediator as elevated pathogenic IFNα, controlled by EC likely by their preserved potent NK-cells and later by other killer cells. Since the earliest days of infection predict outcome genetic or chance events must be key to EC, and since we found no unique immune parameter at the onset, we suggest a chance infection with a lower HIV inoculum. These results offer an additional approach toward functional cure: a judicious targeting of IFNα for all non-EC patients.

## Introduction

Potent anti-retroviral therapy (ART) was chiefly developed through advances in understanding stages and molecular events involved in HIV replication. However, these advances have not led to a “functional cure” in which no further therapy is needed to suppress virus resurgence and immune decline. One approach is development of longer lasting drugs and better delivery. An additional approach is to attempt a greater understanding of HIV pathogenesis. A small group of HIV-infected patients, with undetectable viral load without therapy, known as elite controllers (EC) (< 0.5%), were found by several clinical investigators and championed by Steven Deeks and Bruce Walker (1) who suggested that perhaps the EC status could be mimicked to reach a “functional cure”(2). Individuals with a genotype including HLA_B57+_ (EC_B57+_), which share the Bw4 serotype with the KIR3DL1 allele were found more prone to become EC (3). However, not all EC are HLA_B57+_ (4). Consequently, other mechanisms must contribute to the EC status.

**Extended data Fig. 1:**
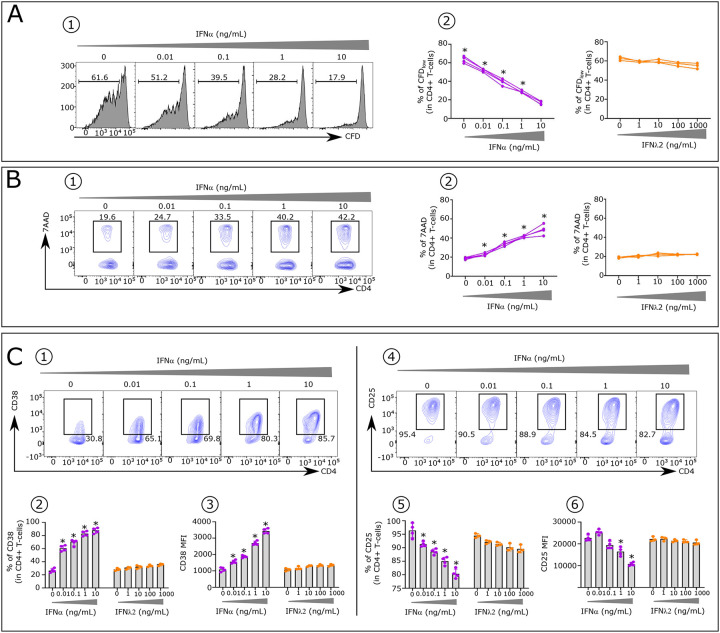
Effect of IFNα and IFNλ2 on stimulated CD4^+^T-cells from HD. CD4^+^T-cells (1.5 × 10^5^ per well) were stimulated with platebound anti-CD3 mAb (pbαCD3) (4 μg/mL) in presence of soluble anti-CD28 mAb (sαCD28) (4 μg/mL) and IL-2 (100 IU/mL) for 4 days. (**A1**) Representative FACS histograms displaying IFNα effect on CD4^+^T-cell proliferation measured by CFD dilution assay. (**A2**) Histograms showing dose–effect of IFNα and IFNλ2 on the frequency of CFD^low^ cells (n=4). (**A1**) Representative FACS histograms displaying IFNα effect on apoptosis of 4 d-stimulated CD4^+^T-cells evaluated by 7-amino-actinomycin D (7-AAD) staining. (**B2**) Histograms showing dose–effect of IFNα and IFNλ2 on the frequency of 7-AAD^+^ cells (n=4). (**C1**) Representative FACS histograms displaying IFNα effect on CD38 expression on 4 d-stimulated CD4^+^T-cells. Histograms showing the IFNα and IFNλ2 dose–effect on (**C2**) the CD38 frequency and (**C3**) the CD38 Mean Fluorescence Intensity (MFI) in CD4^+^ T cells (n=4). (**C4**) Representative FACS histograms displaying IFNα effect on CD25 expression on 4 d-stimulated CD4^+^T-cells. Histograms showing the IFNα and IFNλ2 dose–effect on (**C5**) the CD25 frequency and (**C6**) the CD25 MFI in CD4^+^T-cells (n=4).

Given our long-term studies on the pathogenic (high levels) effects of IFNα in AIDS (5,6), we questioned whether control of the pathogenic effects of IFNα could be a major immune mechanism that is controlled by EC. Indeed, as described in the extended data Table 1, our initial and other reported studies showed that elevated IFNα exerts several pathogenic effects. These occur first at the innate phase of an IR by inhibiting IL-7-induced T-cell proliferation controlling T-cell homeostasis (7); second, at the initiation phase of the adaptive IR by inhibiting proliferation of CD4^+^ T helper cells (8); third, by differentiating T-cells to suppressive IL-10-Tr1 cells (9,10). Systemic IFNα (type I IFN) and mucosal IFN-lambda (IFNλ) (type III IFN) both exert antiviral activities via IFN-stimulated genes (ISGs) (11), albeit with different kinetics of ISGs induction and distinct cell targets expressing specific receptors. IFNAR1 and IFNAR2 are constitutively expressed on virtually all nucleated cells such as CD4^+^T-cells, whereas at homeostasis IFNλ receptors are constitutively expressed on epithelial cells and only in a selected pool of immune cells (12). Consequently, the CD4^+^T-cells are functionally hampered by elevated IFNα at the initiation of the IR adaptive phase, but not by IFNλ even at high levels (extended data Fig. 1). interestingly, a 2023 report shows the efficacy of therapeutic administration of IFNλ in early covid patients (13).

The conclusion that elevated IFNα is a central mediator of HIV pathogenesis is supported by the literature and by results throughout this paper. First, genetic studies have identified polymorphisms in IFN type 1 and type 3 pathways as contributing to variable responses to HIV infection (14). Second, SIV-infected African green monkeys do not express elevated levels of IFNα and avoid development of AIDS (15). By contrast SIV-infected rhesus macaques develop AIDS and have high level of serum IFNα (16). Third, clinical trials results from the EURIS phase 2B placebo control, carried out in 5 hospital centers from 1996–1998 on 240 patients, who had not received combined ART, were based on the induction of anti-IFNα antibodies by an active vaccine approach to lower IFNα. Vaccine responders achieved all study endpoints, including a lower occurrence of HIV-related events, improved CD4^+^T-cell counts and reduced viral load correlating with the rise of anti-IFNα Abs (17). Fourth, we show that IFNα induces a series of immune cell abnormalities in untreated non-EC patients (extended data Table 1 and extended data Fig. 1).

**Extended data Fig. 2:**
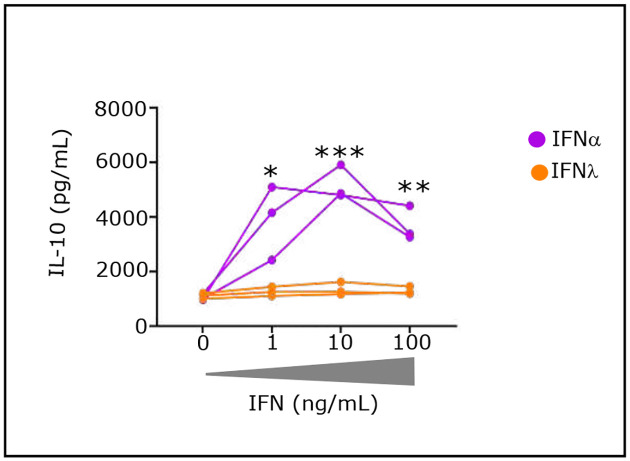
Effect of IFNα and IFNλ2 on the IL-10 secretion by stimulated CD4^+^T-cells from HD. CD4^+^T-cells (1.5 × 10^5^ per well) were stimulated with pbαCD3 (4 μg/mL) in presence of soluble sαCD28 (4 μg/mL) and IL-2 (100 IU/mL) for 4 days. IL-10 levels were quantified by Luminex technology in the 4-d culture supernatant of stimulated CD4^+^T-cells (n=3).

We found that HIV untreated patients (non-EC) have abnormally high concentrations of serum IFNα and a high frequency of phenotypic alterations in all immune cell types. These alterations are known effects of IFNα (extended Fig. 1–2 and extended Table 1). In contrast EC show minimal abnormal immune cell alterations. We further identified potential immune mechanisms enabling each EC to control viral replication by its own immune capacity. Finally, we hypothesized that EC control of IFNα is at least partially circumstantial, namely a fortuitous infection with a low dose of HIV. Since IFNα levels at the primary phase of the acute infection correlate with virus titer, we surmise a vicious circle of HIV → IFNα → more HIV → elevated IFNα → more HIV etc may be the critical factor contributing to loss of virus replication control by non-EC, and in the accompanying manuscript we described the mechanism leading to this vicious circle.

## Results

As detailed in methods, the study compares in untreated non-EC, EC and HD 1) IFNα and IFNλ2 serum concentration, 2) the distribution of immune cell subsets, and 3) the frequency of cell markers associated with immune dysfunction. These include abnormal expression of checkpoint receptors or molecules controlling immune cells function such as 1) inhibitory checkpoint receptors (PD1, CTLA-4) leading cells to an exhaustion stage (18); 2) cell receptor markers of activation/differentiation (CD25/CD38/HLA-DR) associated with chronic immune activation (19); 3) checkpoint receptors controlling pericellular levels of immunosuppressive adenosine (CD39 ectonucleotidases; CD26 harboring adenosine deaminase) (20); 4) apoptotic cell receptors (CD95) and 5) soluble suppressive mediators (IL-10). This phenotypic study focuses particularly on cytotoxic NK-cells, HLA1a-(B)-restricted CD8^+^CTL and HLA1b-(E)-restricted CD8^+^ suppressive cells (CD8^+^supp). Interestingly, CD8^+^supp, which lyse abnormal CD4^+^T-cells expressing HIV-peptides in a HLA-E-restricted presentation, are also present in other chronic inflammatory diseases including autoimmune diseases (21), cancer (22) and other microbial infections (23).

### IFNα is elevated in HIV-infected non-EC and modifies the distribution of blood immune cell types.

**Figure 1:**
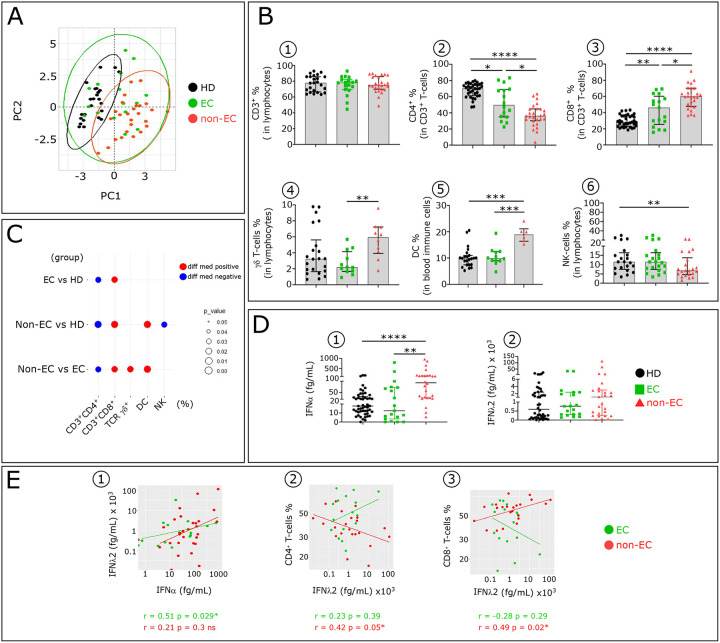
Comparative analysis of major blood immune cell subsets and serum IFNα and IFNλ2 concentration in non-EC, EC and HD. **(A)** Principal component analysis (PCA) of studied participants based on the proportion of different immune cell subpopulations (CD4^+^, CD8^+^ and TCR γδ T-cells, NK and DC), evaluated by flow cytometry. Immune cell profiling was assessed by flow cytometry as depicted in extended data Fig. 3. The first two Principal components (PC1 and PC2) explaining the greatest differences among individuals are represented on a bi-plot. Each point represents one participant, colored by the group they belong to. Each group is outlined by an ellipse representing the 95% confidence interval of the sample groupings. (**B**) Histograms showing distributions of indicated immune cell populations between HD (Black, n=24), EC (green, n=16), and non-EC (red, n=26). (**C**) Balloon-plot summarizing the statistically significant changes in the indicated immune cell populations between EC and HD, non-EC and HD and non-EC and EC. The size of the circle represents the p-value. Red and blue colors show increased or decreased frequencies of the immune cell populations. (**D**) Scatterplots showing IFNα and IFNλ2 concentration in serum from HD (n=51), EC (n=18) and non-EC (n=26). IFNα and IFNλ2 levels were detected by SIMOA. (**E**) Scatterplot showing relationships between IFNα and IFNλ2 serum levels (**E1**), CD4^+^T-cells and IFNλ2 (**E2**), and CD8^+^T-cells and IFNλ2 (**E3**) in EC (n=18) and non-EC (n=26). Correlations were evaluated with Spearman’s rank correlation test. Differences between unpaired samples were performed with Mann-Whitney test. Graph show the median values and p values (*P<0.05, **P<0.01, ***P<0.001, ****P<0.0001)

Blood immune cell types distribution, from non-EC compared to EC and HD, analyzed by Principal Component Analysis (PCA) shows that non-EC and HD cluster separately, while EC overlap both (Fig. 1A). Fig. 1B and 1C document the distinct proportion of circulating immune cell types in non-EC, EC and HD. We found that IFNα, but not IFNλ2, serum concentration was abnormally increased in non-EC compared to HD and the large majority of EC (Fig. 1D). We also found a positive correlation between between IFNλ2 and IFNα serum levels in EC but not in non-EC patients (Fig. 1E1). Moreover, serum IFNλ2 concentration was negatively associated with CD4^+^T-cells, and positively with CD8^+^T-cells frequency in non-EC (Fig. 1E2–3).

### Elevated IFNα induces an abnormal frequency and phenotypic alterations of NK-cells and other innate cell types in non-EC but not in EC.

**Figure 2:**
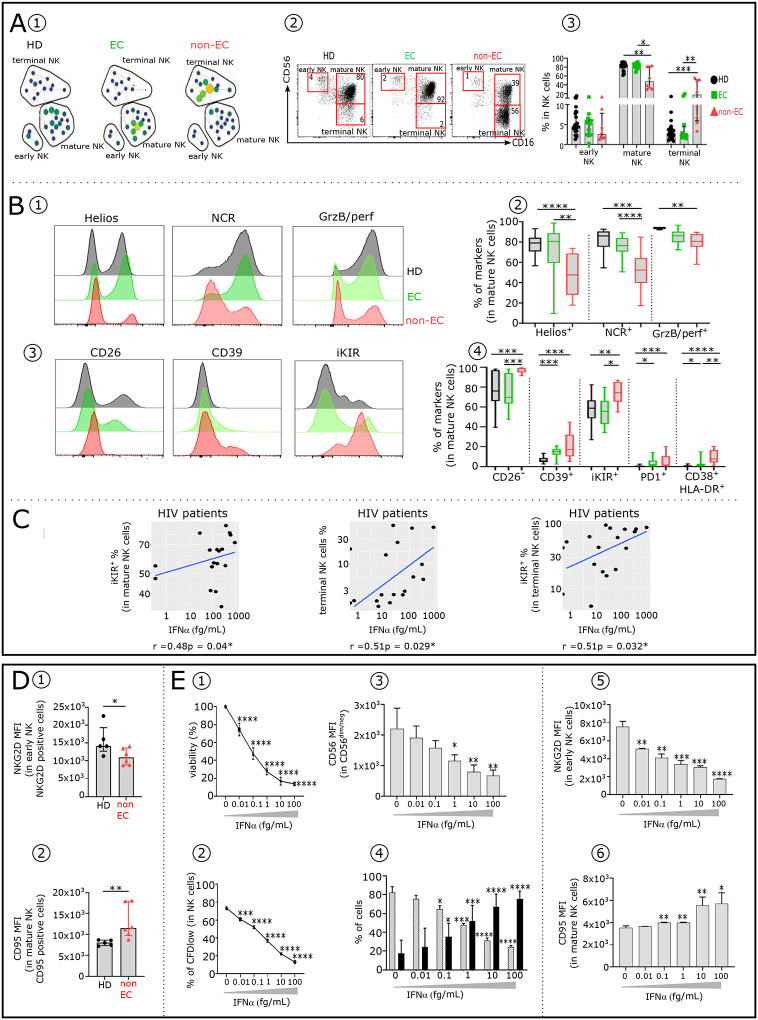
Comparative distribution and immune phenotypic analysis of innate immune NK-cells in HD, EC and non-EC. (**A1**) SPADE tree showing the distribution of the three main NK-cell subsets in HD, EC and non-EC, based on CD56 and CD16 expression levels. Nodes are colored by count. (**A2**) Representative Flow Cytometry plots of NK-cell subsets gated on CD19^−^CD14^−^TCRγδ^−^CD3^−^HLA-DR^−^ cells from the 3 studied groups: early NK (CD56^bright^/CD16^−^), mature NK (CD56^dim^/CD16^+^) and terminal NK (CD56^−^CD16^+^). (**A3**) Frequency of early, mature and terminal NK in each studied group (HD n=22, EC n=12 and non-EC n=8). Profiles displaying the expression level of Helios, NCR (NKp30, NKp44, NKp46), GrzB/perf (**B1)** and CD26, CD39, iKIR (**B3)** on mature NK-cells from HD (black), EC (green) and non-EC (red). (**B2** and **B4**) Box plots displaying the frequency of the indicated markers in mature NK-cells in each studied group. (**C**) Scatterplots showing relationships between IFNα serum level and the frequencies of selected NK-cell subsets. Correlations were evaluated with Spearman’s rank correlation test. Histograms showing the expression level (measured by median fluorescence intensity (MFI) of NKGD2 in early NK-cells (**D1**) and CD95 in mature NK-cells (**D2**) in non-EC (n=6) and HD (n=5). Percentage of viable NK-cells (**E1**) and frequency of CFD^low^ NK-cells (**E2**) after 7 days of culture in presence of increasing doses of IFNα. Histograms showing the expression level of CD56 in CD56^dim/neg^ NK-cells (**E3**), distribution of mature (grey) and terminal NK-cells (black) (**E4**), expression levels of NKG2D in early NK-cells (**E5**) and CD95 in mature NK-cells (**E6**) after 3 days of culture in presence of IFNα. Significance was determined by unpaired Mann-Whitney U test. *P<0.05, **P<0.01, ***P<0.001, ****P<0.0001.

NK-cells are a heterogeneous population of immune cells (24), as shown by spade analysis (Fig. 2A1) and dot plots (Fig. 2A2). As previously reported (25), non-EC had less early and mature NK-cells than HD (2.58% vs 4.61% and 48.1% vs 83.2%), and much more terminal NK-cells (17.91% vs 2.84%) (Fig. 2A3). EC and HD displayed similar proportions of NK-cell subsets. In the mature NK subset, activating markers Helios, NCR and GrzB/perf, were less frequent in non-EC than in EC and HD (Fig. 2B1–2). In contrast, inhibitory Killer-cell immunoglobulinlike receptors (iKIRs), which bind HIV-peptides presented on HLA-1b in activated CD4^+^T-cells as well as inhibitory checkpoint receptors PD1 or CD39, leading cells to an exhaustion-like status (18), were overexpressed in non-EC compared with EC and HD (Fig. 2B3–4). Noteworthy, CD38 and HLA-DR were prevalent in non-EC mature NK-cells. In addition, there was positive correlations between IFNα levels and the frequency of iKIR^+^ mature NK-cells, terminal NK-cells and iKIR^+^ terminal NK-cells (Fig. 2C). This is consistent with IFNα enhancing the development of non-functional NK-cells. Further, indicative of this loss of function among NK-cells in non-EC was the presence of the activation marker NKG2D, only weakly expressed on early NK-cells (Fig. 2D1) and the apoptotic marker CD95 highly expressed on mature NK-cells (Fig.2D2) thereby reducing their cytotoxic activity and increasing their propensity to apoptosis respectively.

To investigate whether elevated IFNα induced these altered NK-cell subset distribution and surface phenotypes, we treated *in vitro* purified NK-cells with increasing dose of IFNα. We found that elevated IFNα inhibited NK-cells viability and proliferation capability in a dose-dependent manner (Fig. 2E1–2). Furthermore elevated IFNα reduced CD56 expression in CD56^dim/neg^NK-cells (Fig. 2E3), leading to a decrease of mature NK-cells frequency and a concomitant increase of terminal NK-cells percentage (Fig. 2E4). Addition of increasing amounts of IFNα in NK-cell culture induced a dose-dependent decrease of NKG2D expression in early (Fig. 2E5) and increase of CD95 expression in mature NK-cells (Fig. 2E6). In summary the NK-cell abnormalities observed in non-EC can be in large part induced by elevated IFNα levels.

As to other innate immune cells, CD11c^−^/CD123^+^ pDC frequency is lower in non-EC than in EC (13.4% versus 32.5 % respectively) (extended data Fig. 4A). This observation was previously made by others (26). γδ T-cells show an abnormally high expression of inhibitory checkpoint receptors in non-EC (extended data Fig. 4B). The expression of these checkpoint receptors is triggered directly by IFNα including CD38 (27), HLA-DR (27) and indirectly by loss of CD26 (28).

### Elevated IFNα alters immune cell homeostasis in non-EC compared to HD and a majority of EC.

Homeostatic disturbances of T-cell subsets after HIV infection are hallmarks of early disease. We investigated the maintenance of CCR7^+^CD4^+^ and CD8^+^T-cells in the 3 groups. As anticipated, a lower frequency of both T-cell subsets was observed in non-EC compared to EC and HD (extended data Fig. 4C1–2). In addition, negative correlations were observed between CD4^+^ and CD8^+^CM T-cell frequency and serum IFNα levels in HIV-patients (extended data Fig. 4C3–4) reflecting the pathogenic inhibitory effects of IFNα on IL-7-induced homeostatic T-cell proliferation (7) occurring in CD4^+^ and CD8^+^CCR7^+^T-cells homing to lymph nodes (29). Interestingly, whereas the frequency of EC_B57+_ CD8^+^CM is similar to HD, the frequency of EC_B57−_ CD8^+^CM is reduced, albeit at a lesser degree than in non-EC (extended data Fig.4C5 and Table 1). The maintenance of T-cell homeostasis observed in EC_B57+_ but not EC_B57−_ (Table 1) may be accounted for by the homology between HLA_B57+_ and NK iKIR (specifically 3DL1 allele) sharing the bw4 serotype. Therefore, iKIR^+^ NK do not recognize HLA_B57_ peptide epitopes, and NK-cells killing is not inhibited. This enables the unaltered EC_B57+_ iKIR^+^ NK cells lysis of infected CD4^+^T-cells resulting in reduction of the viral load and its correlated IFNα production level (30). Consequently, upon TCR stimulation, we observed a reduced percentage of CM T-cells which differentiate into CD4^+^T helper and cytotoxic CD8^+^T-cells in non-EC but not in EC_B57+_.

Besides direct killing of some CD4^+^T-cells by HIV, during the acute phase of infection, early HIV regulatory proteins Nef and Tat are released. Nef reduces cellular HLA-1a expression (^31^), and Tat enhances IFNα production by macrophages (5), thus contributing along with high HIV to the elevated IFNα. This in turn leads to NK-cells phenotypic and functionnal alterations. The outcome is a lack of early lysis of infected CD4^+^T-cells in most HIV-infected patients, as shown here. These immune cell alterations are spared in EC.

### Elevated IFNα in non-EC is associated with a high frequency of IFNα-induced phenotypic abnormalities of CD4^+^ and CD8^+^T-cells.

**Figure 3:**
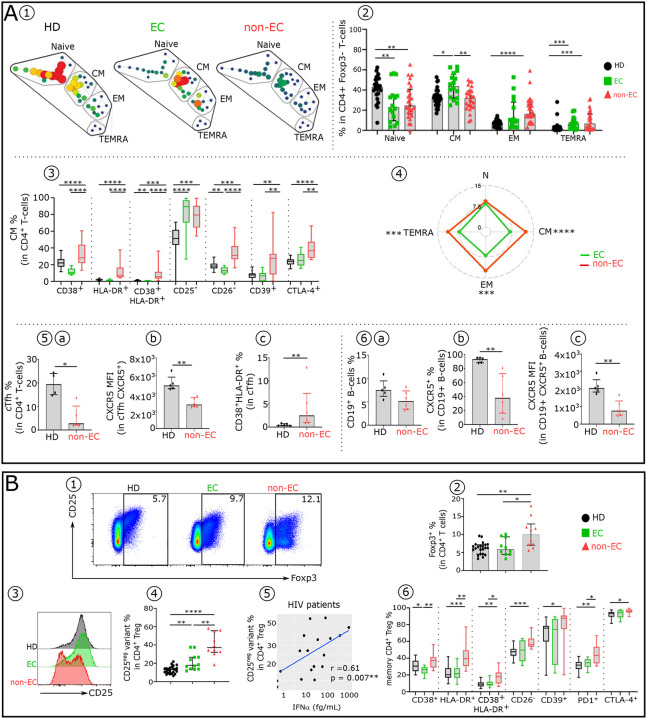
Comparative immune phenotypic analysis of (A) CD4^+^Tconv, CD19^+^B-cells and (B) CD4^+^Treg in non-EC, EC, and HD. (**A1**) SPADE tree showing the distribution of CD4^+^Tconv subsets in HD, EC and non-EC. Nodes are colored by count. CD4^+^Tconv can be classified into four major subsets by their expression of CD45RA and the chemokine receptor CCR7: naïve (CCR7^+^CD45RA^+^); CM (CCR7^+^CD45RA^−^), EM (CCR7^−^CD45RA^−^) and TEMRA (CCR7^−^CD45RA^+^). (**A2**) Frequency of Naïve, CM, EM and TEMRA in each studied group (HD n=24, EC n=16 and non-EC n=23). Boxplots showing the expression of indicated marker in CD4^+^ CM (**A3**) across the groups (HD n=22, EC n=12 and non-EC n=8). (**A4**) Radar chart showing a composite score of phenotypic cell alteration calculated for each CD4^+^Tconv subpopulation in non-EC and EC (see Methods). Frequency of cTfh (**A5a**), expression levels of CXCR5 in cTfh (**A5b**) and frequency of cTfh co-expressing CD38 and HLADR (**A5c**) in non-EC (n=6) and HD (n=5). Proportion of CD19^+^ B-cells (**A6a**), frequency (**A6b**) and expression level (**A6c**) of CXCR5 in CD19^+^B-cells in non-EC (n=6) and HD (n=5). (**B1**) Representative flow cytometry plots of CD25^+^Foxp3^+^ cells within CD4^+^T-cells isolated from HD, EC and non-EC. (**B2**) Histograms showing the frequency of Foxp3 in CD4^+^T-cells, (**B3**) histograms displaying the CD25 expression level in CD4^+^ Foxp3^+^T-cells and **(B4)** the Treg CD25^−^ variant frequency in CD4^+^Foxp3 T-cells in each studied group. **(B5)** Scatterplots showing relationships between frequency of Treg CD25^−^ variant in HIV-infected patients and serum IFNα levels. (**B6**) Proportion of specific functional signaling checkpoint on memory CD4^+^ Treg (CD4^+^ Foxp3^+^CD25^+^CD45RA^−^) of each studied group (HD n=22, EC n=12 and non-EC n=8). Correlations were evaluated using Spearman’s rank correlation test. Significance was determined by unpaired Mann-Whitney U test. *P<0.05, **P<0.01, ***P<0.001, ****P<0.0001.

We first identified the major circulating CD4^+^Tconv (T helper Foxp3^−^) cell subsets at different maturation stages based on their surface expression of CCR7 and CD45RA (32). SPADE analysis done on Tconv (Fig. 3A1) and histograms (Fig. 3A2) show that the frequency of their subtypes vary in both non-EC and EC compared to healthy donors. Both EC and non-EC have significantly lower frequencies of naive CD4^+^T-cells compared with HD. While EC have higher CM and TEMRA, non-EC have higher EM and TEMRA. (Fig. 3A2). We then explored changes in activation/exhaustion phenotypes in CD4^+^T-cell subsets in non-EC compared to EC and HD. The absence (CD26, CD25, CD28) or increase (CD38, HLA-DR,CD39, PD1 and CTLA-4) of these markers are linked to an exhaustion-like state associated with lack of function (18). Each Tconv subset display an altered distinct phenotypic profile in non-EC compared to EC and HD (Fig. 3A3 and extended data Fig.5). CM, EM and TEMRA from non-EC exhibited significantly higher phenotypic alteration scores (see methods) compared to those from EC (Fig. 3A4). These phenotypic abnormalities correspond in large part to the IFNα effects on CD4^+^T-cells in culture (extended data Table 1 and extended data Fig.1). This is consistent with one key HIV pathogenic mediator, namely the effect of elevated IFNα in non-EC. Furthermore, positive correlations are observed between the expression levels of different abnormally expressed markers studied in the CD4^+^CM in non-EC but not in EC (extended data Fig.5e). We then investigated the circulating CXCR5^+^CD45RO^+^CD4^+^ TFH (cTFH) that govern the B-cells hypersomatic mutation and Ig isotype commutation in follicular lymph nodes. Compared with HD, non-EC had lower frequency of cTfh-cells (Fig.3A5a), and, on these cells, they exhibited lower CXCR5 expression level (Fig. 3A5b) and higher coexpression of CD38 and HLA-DR (Fig. 3A5c). In addition, while similar frequency of CD19^+^B-cells was observed between the two studied groups (Fig. 3A6a), non-EC had a reduced percentage of CXCR5^+^B-cells (Fig. 3A6b) and on these cells CXCR5 expression level was lower (Fig. 3A6c). As to the regulatory CD4^+^T-cell subset (CD4^+^Foxp3^+^), their frequency and function were altered in non-EC but not in EC (Fig. 3B). Their proportion was increased (Fig. 3B1–2) and the percentage of non-functional CD25^−^ Treg variant (28) was enhanced (Fig. 3B3–4). Once again, the serum IFNα level significantly correlated with this alteration (Fig. 3B5). In addition, memory CD4^+^Treg phenotypic abnormalities associated with IFNα were multiple in non-EC but minimal in EC. (Fig. 3B6).

**Figure 4:**
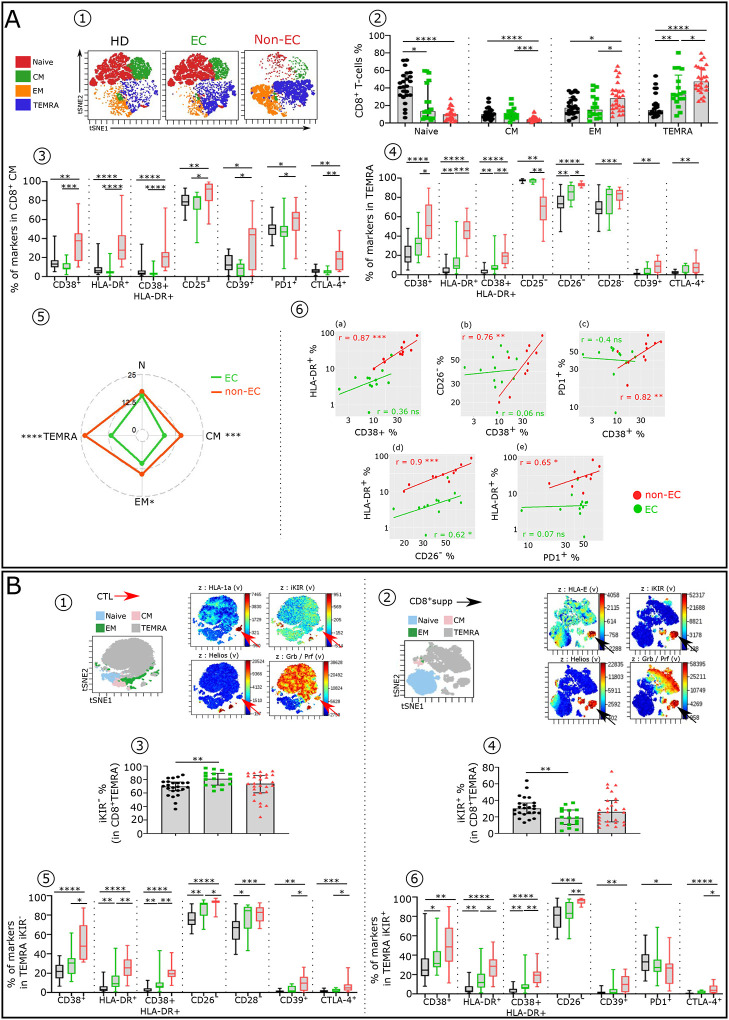
Comparative immune phenotypic analysis of CD8^+^T-cell subsets in non-EC, EC and HD. (**A1**) Representative viSNE plot showing the distribution CD8^+^T-cell subsets, as described above for CD4^+^Tconv in HD, EC, and non-EC. (**A2**) Histograms showing the frequency of Naïve, CM, EM and TEMRA CD8^+^T-cells subsets in each studied group (HD n=24, EC n=16 and non-EC n=23). Boxplots showing the expression of indicated marker in CD8^+^CM (**A3**) and TEMRA (**A4**) across the groups (HD n=22, EC n=12 and non-EC n=8). (**A5**) Radar chart showing a composite score of phenotypic cell alteration calculated for each CD8^+^T-cell subpopulations in EC and non-EC (EC n=12 and non-EC n=8). (**A6**) Scatterplots showing relationships between the expression level of indicated markers in CD8^+^CM (EC n=12 and non-EC n=8). (**B**) ViSNE plot depicting the phenotypic difference between CD8^+^CTL (TEMRA iKIR^−^) (**B1**) and CD8^+^supp (TEMRA iKIR^+^) (**B2**). tSNE plot of CD8^+^T-cell subsets showed in different colors and viSNE projections of expression of indicated markers are shown. Red and black arrows indicate HLA-1a restricted and HLA-E-restricted CD8^+^supp respectively. Histograms showing the frequency of CD8^+^TEMRA iKIR^−^ (**B3**) and TEMRA iKIR^+^ (**B4**) in each studied group (HD n=24, EC n=16 and non-EC n=26). Box plots showing the proportion of specific markers on CD8^+^TEMRA iKIR^−^ (**B5**) and TEMRA iKIR^+^ (**B6**) in each studied group (HD n=22, EC n=12 and non-EC n=8). Correlations were evaluated using Spearman’s rank correlation test. Significance was determined by unpaired Mann-Whitney U test. *P<0.05, **P<0.01, ***P<0.001, ****P<0.0001.

We next performed similar analysis on CD8^+^T-cells in non-EC compared to EC and HD. We examined four well-defined CD8^+^T-cell subsets as above for the Tconv. Distinct distributions of CD8^+^T-cell subsets in the three groups were found, as shown by viSNE analysis (Fig. 4A1) and histograms (Fig. 4A2). Both EC and non-EC had a significantly lower frequency of naive CD4^+^T-cells compared with HD. Non-EC also had lower CM and higher EM and TEMRA than HD and EC (Fig. 4A1–2). As for the Tconv subsets, each CD8^+^T-cell subset had a distinct pattern of phenotypic alterations in non-EC compared to EC and HD. Non-EC CM and TEMRA had a higher phenotypic alteration score than those of EC (Fig. 4A3–5). A large majority of these inhibitory checkpoints receptors are induced by IFNα (extended data Table 1). Furthermore, as described in Fig. 4A6, in non-EC, positive correlations were observed between the expression levels of the various studied markers in CD8^+^CM. CD8^+^ HIV-specific cytotoxic T-cells are comprised of effector HLA-1a-restricted CTL and HLA-E-restricted CD8^+^supp carrying iKIRs, the human analog of Ly49, which characterize murine CD8^+^supp (33,34). HLA-1a-(B) and HLA-1b-(E)-presenting HIV-peptide tetramers recognizing CTL (Fig. 4B1) and CD8^+^supp (Fig. 4B2) respectively. This allowed us to distinguish and phenotypically characterize the two types of cytotoxic CD8^+^T-cells: CTL (CD8^+^, TEMRA, GrzB/perf^+^, iKIR^−^, Helios^−^) and CD8^+^supp (CD8^+^, TEMRA, GrzB/perf^+^, iKIR^+^, Helios^+^). We found similar CTL frequency in EC and non-EC (81.05 % vs 74.15 %) (Fig. 4B3), but the inhibitory checkpoint receptors level was significantly higher in non-EC than in EC and HD (Fig. 4B5), again fitting with the effect of elevated IFNα. This leads to greater CTL activity of EC. As to the CD8^+^supp, non-EC had a slightly higher percentage of CD8^+^supp than EC (25.85% vs 18.95%) (Fig. 4B4) with a more pronounced exhaustion-like stage (CD38, CD39, HLA-DR) or downregulated markers (CD26) (Fig. 4B6), all of which can be induced by IFNα. Of note, similar to the CTL, the CD8^+^supp frequency of EC is also variable from one donor to another.

### The minimal anti-HIV immune cells phenotypic defects and the control of HIV replication in EC is linked to control of IFNα.

**Figure 5:**
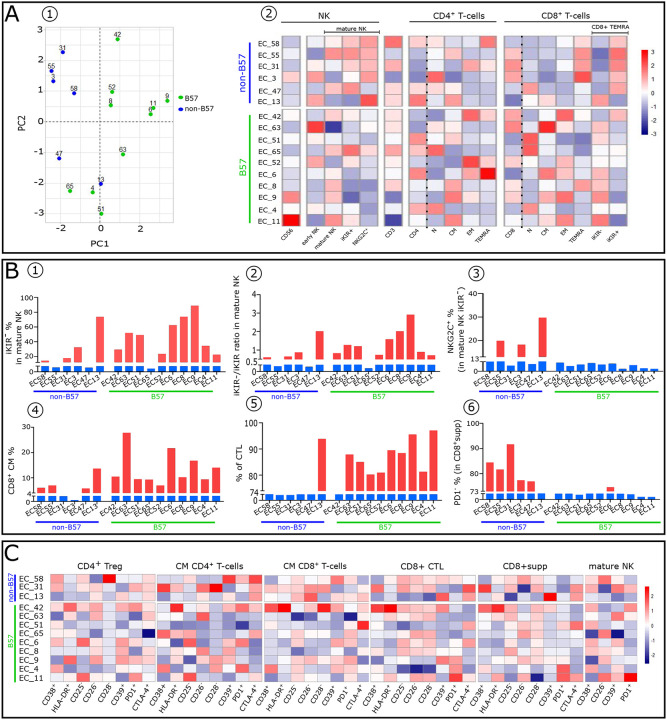
Distinct immune cells phenotypic patterns between non-B57 and B57 EC with a peculiar profile displayed by each patient of these subgroups. (**A1**) PCA scatterplots of CD8^+^T-cell subpopulations frequencies for non-B57 (EC_B57−_) and B57 (EC_B57+_) EC (shown as blue and green dots respectively). (**A2**) Heatmaps representing the distribution of the indicated lymphocytes subsets in EC. Histograms showing frequency and index ratio of indicated subsets in mature NK (**B1–3**) and CD8^+^T-cell compartment (**B4–6**). (**C**) Heatmaps showing the frequency of indicated markers in CD4^+^ Treg, CD4^+^CM, CD8^+^CM, CD8^+^CTL, CD8^+^supp and mature NK-cells.

PCA analysis (Fig. 5A1) and heatmaps (Fig. 5A2) show that EC, whether expressing HLA_B57_ (EC_B57+_) allele or not (EC_B57−_), have a distinct blood immune cell profile variable in each individual. They express a few phenotypic alterations in large part induced by elevated IFNα (Compare to non-EC, Fig. 1D).

To further identify which anti-HIV immune function, uncommonly found in humans, could account for the control of HIV replication in each EC, we investigated which anti-HIV factor(s) known to neutralize circulating virions or to lyse infected CD4^+^T-cells might contribute to the maintenance of the EC status.

Considering the frequency of elevated IFNα -induced immune cell types phenotypic anomalies in non-EC, we examined serum IFNα levels in all patients. The median in non-EC is 95 fg/mL and in EC 18 fg/mL. In our American HIV patient’s cohort, 16 out of 20 non-EC compared to 3 out of 18 EC, showed a higher IFNα level (Table 1). Interestingly, among the 3 EC expressing elevated serum IFNα, EC11 and EC52 compensate their high IFNα, level by the expression of NK-cells activating NKG2C receptor contributing to early lysis of infected CD4^+^T-cells. EC68 cell sample was not available (Table 1). Regarding IFNλ, it is notable that only EC51 had high level compared to other EC (32000 fg/mL vs 1027 fg/mL). pDC frequency were higher in EC compared to other HIV-infected persons (32% versus 14%, Table 1). It is unlikely that greater numbers of pDC be a key immune parameter causing the EC state and in control of elevated IFNα. Rather it is likely that their increase is a consequence of the better control of IFNα in the early days of infection, thereby avoiding its negative impact.

Given that NK-cells act at the onset of infection, and before the virus setpoint, we focused on them in both EC_B57+_ and EC_B57−_. Mature EC_B57+_ NK-cells (9 out of 10 tested) are predominantly iKIR^−^, whereas most mature EC_B57−_ NK-cells are iKIR^+^ (Fig. 5A2 and 5B1). Therefore, these EC_B57+_ NK-cells have the functional capacity to lyse infected CD4^+^T-cells, releasing circulating virions as early as the beginning of the IR. It is notable that one EC_B57−_ (EC13) behaved as an EC_B57+_, but its mechanism is as yet not defined (Table 1). Interestingly, in EC_B57−_ mature NK-cells the expression of NKG2C counterbalance the inhibitory iKIR signals (Fig. 5B3) and allow these NK-cells to kill infected CD4^+^T-cells expressing HIV-peptides in an HLA-E presentation, thus reducing the viral load in EC_B57−_.

The percentage of circulating CD8^+^CM was relatively high in EC_B57+_ (identical to HD median: 10% vs 10%) but reduced in EC_B57−_ albeit higher than in non-EC (median: 6% vs 4%, Table 1) (Fig 5B4 and extended data Fig.4C5). Consequently, the frequency of CTL derived from the differentiation of CD8^+^CM was lower in EC_B57−_ compared to EC_B57+_. It results in higher frequency of CTL than CD8^+^supp in EC_B57+_ (Fig. 5B5 and extended data Fig.6). In contrast, EC_B57−_ have a higher percentage of CD8^+^supp (Fig.5A2 and extended data Fig. 6b) expressing less PD1 (Fig. 5B6). EC_B57−_ (EC13) behaves again as an EC_B57+_. Notably, both subsets are functionally-effective only during adaptive IR and not relevant in the critical earliest days of infection.

Although few immune cell alterations could be detected (Fig. 5C), each EC phenotypic profile was distinct. The majority of these phenotypic alterations, as those found in non-EC, correspond to the direct (HLA-DR, CD38) or indirect (absence of CD26, CD25) effects of IFNα seen in T-cell cultures (extended data Table 1 and extended data Fig. 1). These few but irreversible alterations might occur during the early period of HIV infection in the innate phase of the IR and be augmented by HIV cytopathic effects induced on thymic stromal cells, known to occur during early infection in the innate phase of the IR, and, as reported, also induced by IFNα (35).

## Discussion

We conclude that besides circulating HIV, the key mediator of AIDS development is elevated circulating IFNα. To be a central major mediator of pathogenesis the criterion should be: active at the earliest days of infection, ie., in the innate immune phase, be elevated and circulating, and have broad pathogenic effects. IFNα fits these criteria. In non-EC, it is circulating at elevated levels inducing immune cell type abnormalities linked to poor immune cell function (Fig. 1–5 and Table 1). Correspondingly, in EC the absence of circulating high levels IFNα is associated with minimal immune cell types anomalies.We propose that EC avoid the pathogenic vicious circle of high HIV → high IFNα → more HIV → pathogenic IFNα. These assumptions were based on documented experimental data, as follows:

1) At the onset of infection, the critical role played by NK-cells of EC but not non-EC. These cells lyse target cells prior to the viral setpoint. Thus the NK-cells are likely the most effective immune factor reducing viral load and correlated IFNα levels at the onset. Differences between NK-cells from EC and non-EC (Extended data Fig. 7) reside in their functional capacity. Non-EC NK-cells expressing multiple inhibitory receptors including iKIR and checkpoint receptors such as PD1, are likely not functional (Fig. 2B3–4, Table 1). Moreover the low expression of iKIR in NK-cells from EC_B57+_ enables these unaltered cells to lyse infected CD4^+^T-cells expressing HLA_B57_ restricted HIV epitopes, whereas EC_B57−_ NK-cells expressing iKIR (Fig. 5B1–2), compensate this inhibitory signal by expressing the activating NKG2C receptor (Fig. 5B3) enabling lysis of HLA-E-restricted infected CD4^+^T-cells.

2) The IFNα-induced impairment of IL-7-induced immune cell homeostasis found in non-EC results in reduction of NK-cells and CCR7^+^CD4^+^ and CD8^+^T-cells. As a consequence, populations of helper and cytotoxic T-cells are reduced during the later adaptive IR.

3) The hampered IFNα-induced initiation of adaptive IR in non-EC results in diminished CD4^+^ T-cells activation and proliferation (extended data Table 1).

4) In non-EC, inhibition of CTL and CD8^+^supp function linked to IFNα-induced inhibitory checkpoints results in loss of control of viral replication (Fig.4 and extended data Table 1).

Concerning the possible immune mechanism enabling control of HIV replication possessed by EC postulated by the AAC (2) (Fig. 5), we found that in addition to our hypothesis of a putative lower infectious inoculum, each EC possessed one or more distinct immune mechanisms (Table 1) which could contribute to their avoidance of elevated IFNα levels. These mechanisms include early lysis of infected CD4^+^T-cells by phenotypically-unaltered NK-cells and additionally during the adaptive IR by cytotoxic CTLs and CD8^+^supp. These data prompted us to consider that the EC status does not solely depend on one specific genetic and/or immune cell profile but also on a distinct immune capacity, peculiar to each EC and likely additional circumstantial factors such as an inoculum low size. No doubt that the HLA presentation of HIV-peptides by infected cells, including the known HLA_B57+_ but also HLA-E are early contributing factors for NK lysis and, following the adaptive IR for CTL or CD8^+^supp T-cells. It is also clear that HLA_B57_ genotype is insufficient to account for the EC status, since not all EC are HLA_B57+_ and further HLA_B57+_ is also present in up to 11 percentage of patients with progressive disease, a percentage similar to the uninfected Caucasian population (4). As reported by Altfled et al (36), we also suggest that evasion from cytotoxic NK-cell mediated IR by HIV is far more deleterious for early control of viral replication than CTL and CD8^+^supp, effective only during the adaptive IR. Considering that many immune mechanisms disrupted by HIV due to elevated IFNα in non-EC are avoided by EC (Table 1), we propose that a fortuitous low infectious inoculum is an important contributor to the EC status. This hypothesis has several predictions now under analysis.

This study showed by a comprehensive characterization of the pathogenic levels of IFNα during both anti-HIV innate and adaptive immune reaction : 1) how our data identified elevated IFNα as the key mediator used by HIV to block immune cell attack in non-EC, and 2) why EC, by avoiding this production of elevated IFNα, naturally block HIV replication. Thus, these studies suggest that reducing IFNα production by any acceptable and efficient means could convert non-EC to EC. This proposal is further supported by the experiment carried out on HIV-infected humanized mice showing that anti-IFNα treatment reduced HIV proviral DNA by 14-fold in spleen cells and by 7-fold in bone marrow cells (27). In an accompanying manuscript, we will describe the mechanism for the IFNα inefficient anti-HIV IR promoting its progressive elevation.

## Online Methods

### Human samples.

HD were obtained through Etablissement Français du Sang (EFS, Paris, France). 67 people living with HIV were recruited and subdivided into three group: EC (n=18) were obtained from the NVS cohort (Baltimore), non-EC (n=27) were obtained from NIH (Bethesda n=19) and from the Laboratoire de Référence SIDA (Liège n=11). Patient groups did not significantly differ in terms of age, gender, disease status. All participants or their surrogates provided informed consent in accordance with protocols approved by the regional ethical research boards and the Declaration of Helsinki. Clinical data are indicated in extended data Table 2.

### Sample processing.

Peripheral blood and serum were collected into appropriate tubes. PBMCs were isolated by density gradient centrifugation on Ficoll-Hypaque (Pharmacia, St Quentin en Yvelines, France. PBMCs were stored frozen in liquid.

### Cells culture.

CD4^+^T-cells were isolated from frozen PBMCs. All CD4^+^T-cells were positively selected with a CD4^+^T-cell isolation kit (Miltenyi Biotec, Germany), yielding CD4^+^T-cell populations at a purity of 96–99%. Purified CD4^+^T-cells were stimulated as described previously (28) with 4 μg/mL plate-bound anti-human CD3 (OKT3) mAb (eBioscience, San Diego, CA) and 4 μg/mL soluble anti-human CD28 (CD28.2) mAb (Becton Dickinson) in presence of Recombinant human IL-2 (Proleukine, Chiron, Amsterdam, 100 U/mL) and recombinant human interferon alpha-2a (Roferon-A) and IFNλ2 (Biotechne, UK) at the indicated dose. After five days culture, CD38 and CD25 expression as well as the frequency of 7AAD^+^ cells were measured by flow cytometry on stimulated CD4^+^T-cells (Table S4). NK-cells were isolated from PBMCs. NK-cells were negatively selected with the NK-cell isolation kit (Miltenyi Biotec, Germany), yielding NK-cell populations at a purity of 96–99%. NK-cells were stimulated with IL-15 (Miltenyi Biotec, 10 ng/mL), IL-2 (Proleukine, Chiron, Amsterdam 100 U/mL) and recombinant human interferon alpha-2a (Roferon-A) at the indicated dose. After 3 days of culture, expression of CD56, CD95 and NKG2D was measured by flow cytometry (extended data Table 3a).

### Flow cytometry analysis.

#### MAbs panels, staining.

**Extended data Fig. 3:**
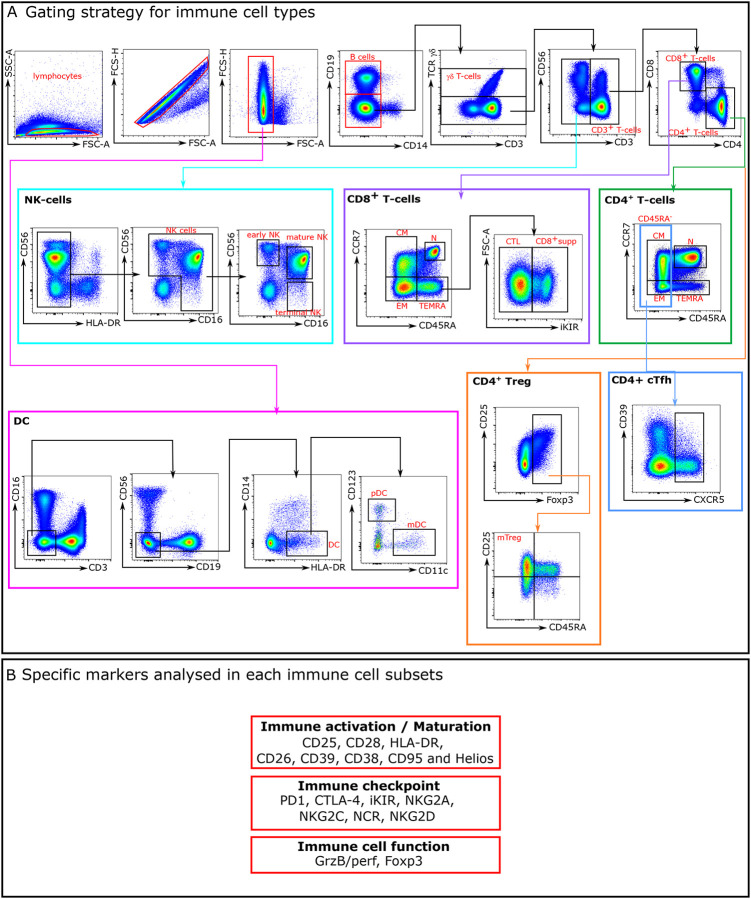
Gating strategy for immune cell types and specific markers analysed in each immune cell subsets. A- Gating strategy for immune cell types. The gating strategy used to identify the main cellular subsets is presented. Arrows are used to visualize the relationships across plots, and numbers are used to call attention to populations described here. After doublets and dead cells were excluded, lymphocytes were gated based on FSC-A/SSC-A properties. From the CD14^−^CD19^−^ lymphocyte gate, the following populations were identified: CD3^+^TCRγδ^+^, TCRγδ^−^ were subdivided in CD3^−^ and CD3^+^T-cells. NK-cells were defined as CD3−TCRγδ−HLA-DR− and classified as early NK (CD56^+^CD16−), mature NK (CD56^+^CD16^+^), and terminal NK (CD56−CD16^+^) cells. The CD3^+^TCRγδ− population was divided in CD4^+^ and CD8^+^ T-cells. In CD4^+^T-cells subpopulation, CCR7^+^ and CD45RA^+^ were used to further classify these cells in four subpopulations: N (CCR7^+^CD45RA^+^), CM (CCR7^+^CD45RA^−^), EM (CCR7^−^CD45RA^−^) and TEMRA (CCR7^−^CD45RA^+^). Tregs were identified from the CD4^+^ population using Foxp3 expression. Foxp3^+^ cells were classified in naïve and memory Treg cells using CD45RA and CD25 markers. CD45RA^−^CD25^+^ represent the memory Treg cells population. As for CD4^+^T^_^cells, CD8^+^T^_^cells were classified using CD45RA and CCR7 markers: four populations were identified: N (CCR7^+^CD45RA^+^), CM (CCR7^+^CD45RA^−^), EM (CCR7^−^CD45RA^−^) and TEMRA (CCR7^−^CD45RA^+^). Among TEMRA CD8^+^T-cells, we distinguished two cytotoxic subpopulations: iKIR^+^ (CD8^+^supp) and iKIR^−^ (CTL). Dendritic cells (DCs) were identified by gating on CD3^−^CD19^−^CD56^−^CD14^−^HLA^−^DR^+^ and from there CD123^+^CD11c^−^ (pDCs) and CD11c^+^CD123^−^ mDCs were identified. B- Specific markers analysed in each immune cell subsets.

Immunophenotypic studies were performed on frozen samples, using up to 23-colours flow cytometry panels. See extender data Table 3 for antibody panel information. The list of mAbs used are detailed in gating strategy we used to identify the immune cell subtypes and their respective subsets are represented in extended data Fig. 3. Antibody titration was performed to choose the concentration that provided the maximal brightness of the positive cell population and the lowest signal for the negative cell population. Approximately 1 × 10^6^ to 5 × 10^6^ frozen PBMCs were used per patient per stain. Staining was performed as described previously (28) except for the panel using HLA-E- or HLA-A*02-pentamers (Pro-immune). Pre-incubation with a blocking anti-CD94 mAb (clone HP-3D9, 5 μg/mL, BD Biosciences) was performed to completely abrogate the non-specific staining of CD94^+^/NKG2^+^ T cells by HLA-E-pentamers before the staining with the other antibodies. Cells were acquired on Cytek Aurora flow cytometer. Data were analysed using FlowJo software (FlowJo, LLC). Flow cytometry data were identified as the proportion (%) of cells expressing the marker, and protein levels as MFI. Unsupervised analyses were performed using cytobank software and R studio software.

#### 7-AAD (7-amino-actinomycin D) staining.

Apoptosis of stimulated CFD-labeled CD4^+^T-cells was determined using the 7-AAD assay (1). Briefly, cultured cells were stained with 20 μg/mL nuclear dye 7-AAD (Sigma-Aldrich) for 30 min at 4 °C. FSC/7-AAD dot plots distinguish living (FSC^high^/7-AAD−) from apoptotic (FSChigh/7-AAD+) cells and apoptotic bodies (FSClow/7− AAD+) and debris ((FSClow/7-AAD−). Living cells were identified as CD3^+^7-AAD−FSC^+^ cells (37).

#### CellTrace violet staining.

CD4^+^T-cells and NK-cells were stained with 1 μM dye (CellTrace violet; Molecular Probes/Invitrogen) in PBS for 8 min at 37°C at a concentration of 1 X 10^6^ cells/mL. The labelling was stopped by washing the cells twice with RPMI-1640 culture medium containing 10% FBS. The cells were then re-suspended at the desired concentration and subsequently used for proliferation assays.

### Composite cell phenotypic alteration score.

We generate a cumulative phenotypic score for each T-cell subsets. The frequency of the following markers was used to calculate the score: CD25^−^,CD26^−^;HLA-DR^+^, CD38^+^, CTLA-4^+^,CD28^−^, PD1^+^ and CD39^+^. It is calculated as the sum of the ratio of the expression level of each marker to the average expression level of the corresponding marker in the HD.

### Cytokines quantification.

Serum IFNα and IFNλ2 levels were determined using Simoa cytokine assays (references 100860 and 101419 respectively). Van der Sluis et al showing that HIV infection of co-cultures of CD4^+^T-cells and pDCs enhanced mRNA expression of IFNλ2 and not IFNλ1 or IFNλ3, we focused only on serum IFNλ2 levels (38). IL-10 in 4 day-cell culture supernatants was determined by Luminex technology (human custom Procarta Plex, Invitrogen)

### Statistical analyses.

Statistical significance of differences between groups was assessed using the unpaired nonparametric Mann-Whitney. Non-parametric, paired Wilcoxon tests were used for paired data. Correlations were assessed by the nonparametric Spearman test. Analyses were performed with GraphPad-Prism, and R. Two-sided P value less than .05 was considered statistically significant (ns: nonsignificant; *P < .05; **P < .01; ***P < .001; ****P < .0001).

## Extended Data

**Extended data Table 1:** IFN type I effects on immune cell subtypes and its correlation with suppressive cytokines.

A-Known potential pathologic effects during chronic viral infection of IFN type I			
Cellular mechanism	Cell type	effects	References
**Homeostasis**	T-cells	Inhibits IL-7 signaling pathway	(39,7)
**Proliferation**	T-cells	Inhibits T-cell proliferation	(40)
**Hyper-activation**	T-cells	Increases of CD38, HLA-DR	(41,42,27,43)
**Loss of function**	T-cells	Enhances PD-1 expression	(44,43,45,46)
		Decreases CD25 expression	(47)
		Decreases CD28 expression	(48)
	DCs and other APCs	Upregulates PD-L1, IL-10	(49,50)
**Apoptosis**	T-cells	Enhances expression of CD95, Bak, TRAIL	(51,52,53)
	DCs and other APCs	Upregulates and pro-apoptotic molecules	(54,55)
**Involution**		Induces thymic involution	(35,56)

B-Known correlation between IFN type I and immune suppressive cytokines		
Suppressive cytokine	effects	References
**TGF-β1**	IFNα enhances TGF-β1 signaling pathways	(57)
**IL-10**	IFNα promotes CD4^+^T-cells differentiation into suppressive IL-10 secreting Tr1 cells	(9,10)

**Extended data Table 2:** Clinical data for HIV patients

*Table 2a : EC patients*								
EC	HIV dx	Sample date	CD4 at sample date	VL	HLA B57	Sex	Race	HIV risk factor
3	1991	10/12/012	1140	334	−	M	AA	IDU
4	1993	22/08/2011	952	<40	+	F	AA	HS
6	1992	31/05/2011	1889	<75	+	F	AA	IDU
8	1989	01/02/2010	1018	<40	+	M	AA	IDU
9	2003	25/09/2014	496	<40	+	M	AA	IDU
11	1997	10/72/011	917	<40	+	M	AA	IDU
13	1993	21/02/2012	864	<40	−	M	AA	IDU
31	1992	23/05/2012	587	<48	−	M	AA	MSM
32	2000	23/05/2012	643	<48	−	F	AA	IDU
42	1990	13/11/2013	731	<220	+	M	AA	IDU
47	2007	01/12/2013	891	<20	−	F	AA	IDU
51	2007	20/10/2014	1250	<40	+	F	AA	HS
52	1992	01/08/2012	340	<20	+	M	AA	IDU
55	1994	01/02/2013	482	50	−	M	AA	IDU
58	1991	01/03/2013	1745	59	−	F	AA	HS
63	1988	24/01/2014	632	32	+	F	AA	IDU
65	1991	01/06/2013	1792	<48	+	M	AA	HS
68	2002	01/07/2010	584	169	+	F	AA	HS

*Table 2b : non-EC patients*				
Untreated patients study_ID	sample date	CD4 at sample date	VL	Origin
**1**	03/11/2004	506	23808	NIH (Bethesda)
**2**	22/04/2004	516	58059	
**3**	01/10/2005	230	153719	
**4**	27/09/2005	327	132773	
**5**	19/06/2006	419	1715	
**6**	04/10/2007	513	12015	
**7**	13/08/2007	447	21701	
**8**	13/03/2008	319	4785	
**9**	05/11/2011	756	7011	
**SS_20**	08/10/2005	445	407	
**SS_21**	11/10/2009	292	3012	
**SS_22**	10/07/2010	477	29257	
**SS_23**	07/22/08	639	<50	
**SS_24**	11/17/2008	267	26059	
**SS_25**	03/23/2006	470	5384	
**SS_26**	11/07/2012	740	11417	
**SS_27**	01/28/2014	344	63954	
**SS_28**	11/04/2010	872	7180	
**SS_29**	12/03/2002	196	17809	
**AZT70934**	12/03/2021	470	2480	Laboratoire de Référence SIDA (Liège)
**AZT71312=1373**	12/03/2021	100	24800	
**AZT_71320_AFCHM_6**	12/08/2020	139	32800	
**AZT_71333_KWGIF_7**	12/09/2021	356	9790	
**AZT_71374_DIAMM_8**		390	588000	
**AZT_71391_GAFUM_9**	02/24/2021	1064	14700	
**AZT71422**	03/05/2021	30	850000	
**AZT_72336_GEMAM_11**		20	824	
**AZT_72498_YOLEF_12**		280	82700	
**AZT_72607_CHUIN**	03/16/2021	577	157000	
**AZT_72644_NGNNF**	03/18/2021	180	73700	

**Extended data Table 3:** mAb list

*Table 3a: mAb lis for the immune cells culture phenotype*			
Markers	Fluorochrome	Clone	Origin
**CD3**	AF532	UCHT1	Invitrogen
**CD4**	BV510	RPA-T4	BioLegend
**CD8**	BV750	RPA-T8	BioLegend
**CD25**	BV786	M-A251	BD Biosciences
**CD38**	PerCPeF710	HB7	Invitrogen
**CD56**	BV711	HCD56	BioLegend
**CD16**	Ef450	eBioCB163	Invitrogen
**NKG2D**	PE	AD11	BD Biosciences
**CD95**	BV421	DX2	BD Biosciences
**7AAD**	7AAD		Sigma Aldrich

*Table 3b: mAb lis for the immune cells panel*				
	Markers	Fluorochrome	Clone	Origin
**Immune cell types**	CD3	AF532	UCHT1	Invitrogen
	CD4	BV510	RPA-T4	BioLegend
	CD8	BV750	RPA-T8	BioLegend
	CD56	BV711	HCD56	BioLegend
	CD16	Ef450	eBioCB163	Invitrogen
	TCR-γδ	BV480	11F2	BD Biosciences
	CD19	BV750	HIB19	Biolegend
	CD14	AF647	MOP9	BD Biosciences
	CD123	PerCpCy5.5	7G3	BD Pharmingen
	CD11c	BV605	3.9	BioLegend
**Immune Activation / Maturation**	CD45RA	FITC	REA562	Miltenyi Biotech
	CCR7	BV421	G043H7	Biolegend
	CD28	APC-R700	CD28.2	BD Biosciences
	CD25	BV786	M-A251	BD Biosciences
	HLA-DR	APCCy7	1243	Biolegend
	CD26	PE	BA5b	Biolegend
	CD39	PeCy7	A1	BioLegend
	CD38	PerCPeF710	HB7	Invitrogen
**Immune checkpoint**	PD1	BV650	EH12.2H7	BioLegend
	CTLA-4	PeCy5	BNI3	BD Biosciences
	KIR2DL1	APC	REA284	Miltenyi Biotech
	KIR3DL1/DL2	APC	REA970	Miltenyi Biotech
	KIR2DL2/DL3	APC	DX27	Miltenyi Biotech
	KIR2DL5	APC	REA955	Miltenyi Biotech
**T-cell function**	Foxp3	PeCF	236A/E7	BD Biosciences
**Viability**	Zombie	NIR		Biolegend

*Table 3c: mAb lis for CD8*^+^ *T-cells panel*				
	Markers	Fluorochrome	Clone	Origin
**Immune cell types**	CD3	AF532	UCHT1	Invitrogen
	CD4	BV510	RPA-T4	BioLegend
	CD8	BV570	RPA-T8	BioLegend
	CD56	APC-Cy7	HCD56	BioLegend
**Immune Activation / Maturation**	CD45RA	BV421	HI100	BioLegend
	CCR7	BV785	G043H7	BioLegend
	CD28	APC-R700	CD28 .2	BD Biosciences
	Hélios	PE-Dazzle594	22F6	BioLegend
**Immune checkpoint**	NKG2A	PE-Vio770	REA110	Miltenyi Biotech
	KIR2DL1	APC	REA284	Miltenyi Biotech
	KIR3DL1/DL2	APC	REA970	Miltenyi Biotech
	KIR2DL2/DL3	APC	DX27	Miltenyi Biotech
	KIR2DL4	APC	REA768	Miltenyi Biotech
	KIR2DL5	APC	REA955	Miltenyi Biotech
	NKG2C	VioBright	REA205	Miltenyi Biotech
	Nkp30	PE-Cy5	Z25	Beckman Coulter
	Nkp44	PE-Cy5	Z231	Beckman Coulter
	Nkp46	PE-Cy5	BAB281	Beckman Coulter
	PD1	BV650	EH12.2H7	BioLegend
**Viability**	zombie	NIR		BioLegend

*Table 3d: mAb lis for cytotoxic CD8*^+^ *T-cells panel*				
	Markers	Fluorochrome	Clone	Origin
**Immune cell types**	CD45	PerCP	HI30	BioLegend
	CD3	AF532	UCHT1	Invitrogen
	CD4	BV510	RPA-T4	BioLegend
	CD8	BV570	RPA-T8	BioLegend
	CD56	APC-Cy7	HCD56	BioLegend
**CD8+ cytotoxic T-cells**	HLA1-a	BV711	pentamer	Proimmune
	HLA-E	PE	E*01 :01	Proimmune
**Immune Activation / Maturation**	CD45RA	BV421	HI100	BioLegend
	CCR7	BV785	G043H7	BioLegend
	CD28	APC-R700	CD28 .2	BD Biosciences
	Helios	PE-Dazzle594	22F6	BioLegend
	Nkp30	PE-Cy5	Z25	Beckman Coulter
	Nkp44	PE-Cy5	Z231	Beckman Coulter
	Nkp46	PE-Cy5	BAB281	Beckman Coulter
**Immune checkpoint**	NKG2A	PE-Vio770	REA110	Miltenyi Biotech
	KIR2DL1	APC	REA284	Miltenyi Biotech
	KIR3DL1/DL2	APC	REA970	Miltenyi Biotech
	KIR2DL2/DL3	APC	DX27	Miltenyi Biotech
	KIR2DL5	APC	REA955	Miltenyi Biotech
	NKG2C	VioBright	REA205	Miltenyi Biotech
	PD1	BV650	EH12.2H7	BioLegend
**Imune cell function**	GrzB/perf	PerCP-cy5.5	QA16A02/B-D48	Biolegend
**Viability**	zombie	NIR		BioLegend

**Extended data Fig. 4:**
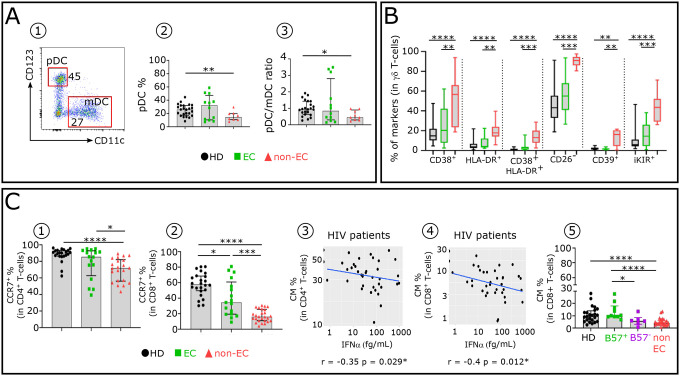
Immune cell types of non-EC but not of EC involved in the innate phase of an anti-HIV IR exhibit distinct altered pattern linked to elevated IFNα. **(A)** Representative dot plot showing how to distinguish pDC (CD123^+^CD11c^−^) and mDC (CD123^−^CD11C^+^) subsets within the HLA-DR^+^lin^–^ population in HD **(A1)**. Histograms showing the frequencies of pDC **(A2)** and the pDC:mDC ratio **(A3)** across the groups (HD n=22, EC n=12 and non-EC n=8). (**B**) Specific markers proportion on TCR γδ T-cells of each studied group (HD n=22, EC n=12 and non-EC n=8). (**C**) Histograms showing the frequencies of CCR7 in CD4^+^ (C1) and CD8^+^ (C2) T-cells across the groups (HD n=22, EC n=12 and non-EC n=26). Scatterplots showing relationships between the frequencies of CD4^+^CM (C3) and CD8^+^CM (C4) T-cells with IFNα levels in HIV-1-infected patients. (C5) Histograms showing the frequencies of CD8^+^CM in HD (n=22), HLA-B57^+^ EC (n=10), HLA-B57^−^ EC (n=6) and non-EC (n=26). Significance was determined by unpaired Mann-Whitney U test. *P<0.05, **P<0.01, ***P<0.001, ****P<0.0001.

**Extended data Fig. 5:**
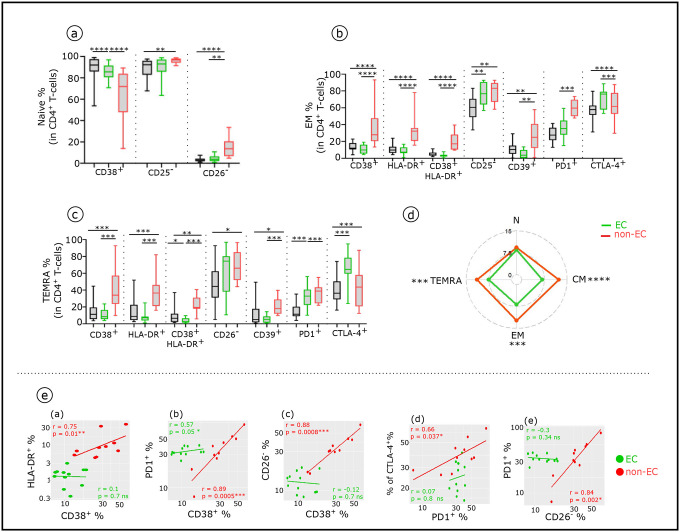
Frequency and phenotypic alterations of CD4^+^T-cell subsets in non-EC and EC Boxplots showing the expression of indicated marker in CD4^+^Naïve (**a**), EM (**b**) and TEMRA (**c**) across the groups (HD n=22, EC n=12 and non-EC n=8). (**d**) Radar chart showing a composite score of phenotypic cell alteration calculated for each CD4^+^Tconv subpopulation in non-EC and EC (see Methods). (**e**) Scatterplots showing relationships between the expression level of indicated markers in the CD4^+^CM subsets (EC n=12 and non-EC n=8). Correlations were evaluated with Spearman’s rank correlation test. Significance was determined by unpaired Mann-Whitney U test. *P<0.05, **P<0.01, ***P<0.001, ****P<0.0001.

**Extended data Fig. 6:**
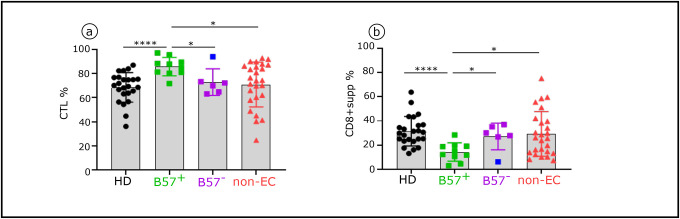
Frequency of CTL and CD8^+^supp in EC-B57^+^, EC-B57^−^, non-EC and HD. Histograms showing distributions of CTL and CD8^+^supp between HD (Black, n=24), EC-B57^+^ (green, n=10), EC-B57^−^ (purple and blue, n=6) and non-EC (red, n=26). One EC-B57^−^ (EC13) in blue behaves as an EC-B57^+^. Significance was determined by unpaired Mann-Whitney U test. *P<0.05, **P<0.01, ***P<0.001, ****P<0.0001.

**Extended data Fig. 7:**
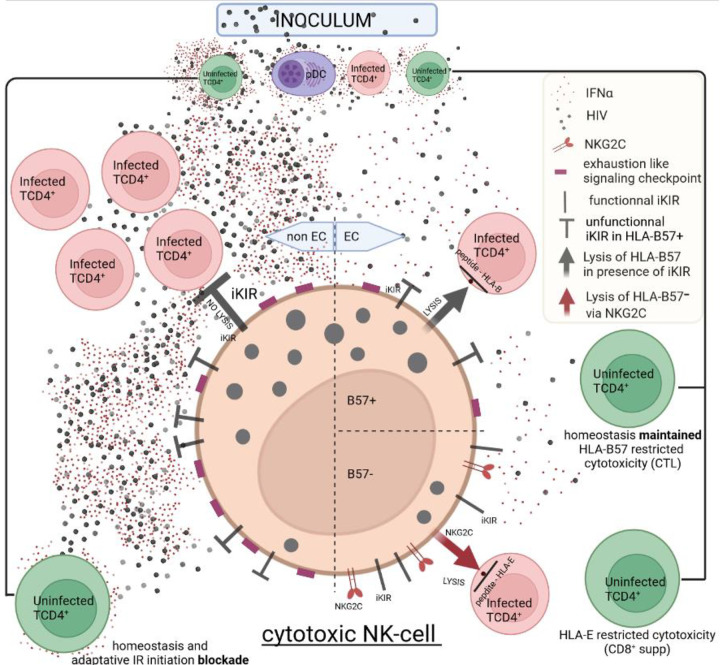
NK-cell cytotoxic activity varies in HIV-infected patients according to their status. 1-Left: in non-EC, NK-cells are inactive given the high frequency of their inhibitory checkpoints leading to a state of exhaustion in large part induced by IFNα. 2-Right and up: in HLA-B57^+^ EC NK-cells, which possess negligible iKIR and further express negligible inhibitory receptors, lyse infected target cells expressing HLA-B restricted HIV-peptides. 3-Right and down: in HLA-B57^−^ EC NK-cells, which express iKIR, but have negligible inhibitory checkpoints, express the activating NKG2C receptor, counterbalancing the iKIR signaling, and can thereby kill infected cells carrying HIV-peptides in an HLA-E restriction. Figure created with Biorender.

## Figures and Tables

**Table 1:** Contribution of immune mechanisms to the Elite Controler status

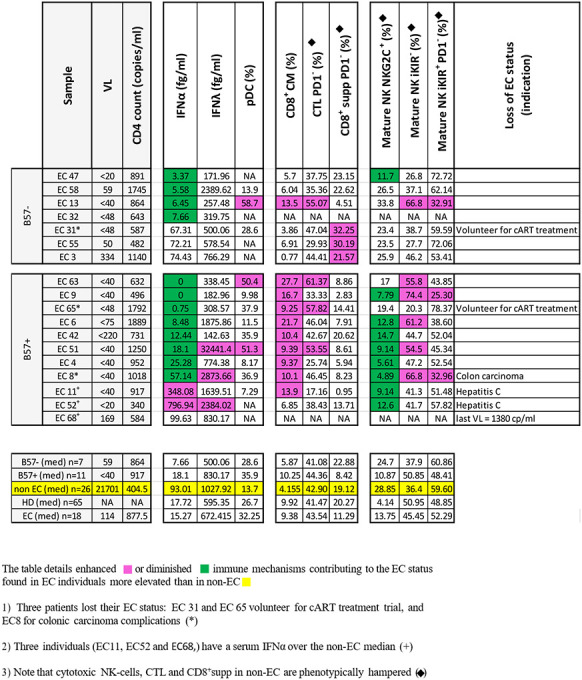

## Abbreviations:

ADO: Adenosine
ATP: adenosine triphosphate
AMP: Adenosine monophosphate
cART: combined antiretroviral therapy
CD8^+^supp: cytotoxic CD8^+^ suppressive T-cell
CM: Central Memory
CTL: CD8^+^ cytotoxic T lymphocyte
DC: Dendritic cells
EC: Elite Controller
EM: Effector Memory
HD: Healthy Donor
IR: Immune Reaction
mRNA: messenger ribonucleic acid
NK: Natural Killer
TEMRA: Terminal Differentiated Effector Memory
